# Photon Upconversion in Small Molecules

**DOI:** 10.3390/molecules27185874

**Published:** 2022-09-10

**Authors:** Dorota Bartusik-Aebisher, Mateusz Mielnik, Grzegorz Cieślar, Ewa Chodurek, Aleksandra Kawczyk-Krupka, David Aebisher

**Affiliations:** 1 Department of Biochemistry and General Chemistry, Medical College of The University of Rzeszów, University of Rzeszów, 35-959 Rzeszów, Poland; 2 English Division Science Club, Medical College of The University of Rzeszów, University of Rzeszów, 35-959 Rzeszów, Poland; 3 Department of Internal Medicine, Angiology, and Physical Medicine, Center for Laser Diagnostics and Therapy, Medical University of Silesia in Katowice, 41-902 Bytom, Poland; 4 Faculty of Pharmaceutical Sciences in Sosnowiec, Medical University of Silesia in Katowice, 40-055 Katowice, Poland; 5 Department of Photomedicine and Physical Chemistry, Medical College of The University of Rzeszów, University of Rzeszów, 35-959 Rzeszów, Poland

**Keywords:** photon, upconversion, nanomaterials

## Abstract

Upconversion (UC) is a process that describes the emission of shorter-wavelength light compared to that of the excitation source. Thus, UC is also referred to as anti-Stokes emission because the excitation wavelength is longer than the emission wavelength. UC materials are used in many fields, from electronics to medicine. The objective of using UC in medical research is to synthesize upconversion nanoparticles (UCNPs) composed of a lanthanide core with a coating of adsorbed dye that will generate fluorescence after excitation with near-infrared light to illuminate deep tissue. Emission occurs in the visible and UV range, and excitation mainly in the near-infrared spectrum. UC is observed for lanthanide ions due to the arrangement of their energy levels resulting from f-f electronic transitions. Organic compounds and transition metal ions are also able to form the UC process. Biocompatible UCNPs are designed to absorb infrared light and emit visible light in the UC process. Fluorescent dyes are adsorbed to UCNPs and employed in PDT to achieve deeper tissue effects upon irradiation with infrared light. Fluorescent UCNPs afford selectivity as they may be activated only by illumination of an area of diseased tissue, such as a tumor, with infrared light and are by themselves atoxic in the absence of infrared light. UCNP constructs can be monitored as to their location in the body and uptake by cancer cells, aiding in evaluation of exact doses required to treat the targeted cancer. In this paper, we review current research in UC studies and UCNP development.

## 1. Introduction

Photon upconversion (UC) through the use of lanthanide-doped materials has been the subject of an increasing amount of research in materials chemistry and physics for over 50 years. The attraction of this field is the ability to generate photons at shorter wavelengths than the excitation wavelength after laser stimulation. Despite its potential utility in many applications, photon conversion has been studied primarily in bulk glass or crystalline materials. This situation changed radically in the mid-2000s, with extensive research into upconversion nanomaterials. As a unique class of optical materials, upconvertable nanomaterials have useful applications ranging from lighting to volumetric 3D displays to photovoltaics. In particular, upconversion nanocrystals have proved to be valuable as luminescent markers for chemical and biological detection, with marked improvements in the sensitivity and versatility of the sensors.

**Figure 1:**
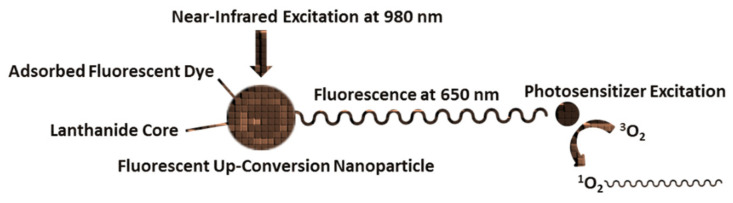
Scheme of UCNP structure.

Photodynamic therapy (PDT) is a cancer treatment in which the UC phenomenon can help to achieve more efficient treatment due to deeper-delivery upconversion nanoparticles (UCNPs) in tissue. PDT uses photogenerated reactive oxygen species (ROS) to damage targeted cells. For generation of ROS, a PDT employs photosensitizers (PS) that are excited by external illumination provided by visible light at power levels that do not damage healthy tissue. The primary ROS generated is singlet oxygen (^1^O_2_), which reacts with cell molecules, ultimately resulting in tissue damage and cell death. The mechanism of ^1^O_2_ formation in this system is energy transfer from excited PS to ground-state oxygen. Other ROS, such as superoxide and hydroxyl radical, are also generated to a much lesser extent and can also be toxic to cells. Singlet oxygen is a short-lived ROS (<1 μs in the cell), thus a requirement is to generate ^1^O_2_ within a distance of ca. 20 nm from targets within a cell. PS tends to accumulate on or internalize in targeted cells and in healthy tissue to some extent. After delivery to targeted cells, the PS is remotely activated by an external light source producing toxic ROS in the presence of oxygen. This methodology imparts spatial and temporal selectivity in treatment, as the PS localized on targeted cells is activated by selectively illuminating the region of diseased tissue, resulting in necrosis and/or apoptosis [1,2]. PS cannot generate ROS in the absence of light, and ideally are atoxic to heathy cells in the dark. Research in PDT is ongoing, as this approach has yet to reach its full clinical potential, although several PS are approved for use [2]. Current limitations in PDT include tissue irradiation with visible wavelengths that have a shallow tissue-penetration depth. Limitations may be addressed in the design of PDT systems that can fluoresce visible light in deep tissue. Complementary research must also address facets of PDT that are diagnostic in nature, such as imaging PS delivery and measuring the amount of singlet oxygen produced on or within a cell and the number of ^1^O_2_ molecules required to kill a single cell. PDT is a subject of intense research, although a few laboratory PDT systems have made the transition to clinical use [2]. In the USA, several PS have been approved by the Food and Drug Administration and are in use, with many currently in clinical trials [2]. UCNPs are examples of small compounds that—upon excitation of the lanthanide core with 980 nm light—can fluoresce following Förster resonance energy transfer (FRET) from the core to the PS (Figure 1). An optimal UCNP diameter for FRET has recently been reported [1]. The fluorescence generated can then be used to excite nearby ^1^O_2_ photosensitizers that are clinically approved for deep-tissue PDT.

Recently, many examples of UCNPs with covalently attached PS have been designed [1,2,3,4,5,6,7,8,9,10,11,12,13,14]. The main approach in PDT research is to adsorb anionic PS, such as rose bengal to surface-positive lanthanide UCNPs, which, following FRET-driven dye fluorescence, excite nearby ^1^O_2_ PS from an initial pulse of near-infrared light that can penetrate tissue up to 2 cm deep. The UCNPs described here are considered as nanotransducers as they absorb near-infrared light (NIR) and emit visible light (fluorescence) at wavelengths absorbed by free PS [5]. Rose bengal is adsorbed to lanthanide UCNPs for generation of ca. 650 nm FRET-driven fluorescence. PS that can generate ^1^O_2_ via excitation at ca. 650 nm, such as methylene blue (as a model PS), along with chlorin-based clinically approved PS, such as photochlor (HPPS) and talaporfin sodium (mono-L-aspartyl chlorin e6 (NPe6)), was employed to evaluate photodynamic action as a result of UCNP fluorescence.

## 2. Photon Upconversion Phenomenon

Nanotechnology is the science dealing with the design, synthesis, and application of structures and materials whose minimum one dimension is in the range from 1 to 100 nm. Nanotechnology can have a significant impact on solving contemporary problems, as presented by the chemist Richard Smalley [3], winner of the Nobel Prize (1996 discovery of fullerenes) and the chief spokesman of the National Nanotechnology Initiative in 2003, in particular through the production of professional materials, fuel cells, solar cells, batteries, catalysts, analytical equipment, sensors, and biosensors [4].

**Figure 2:**
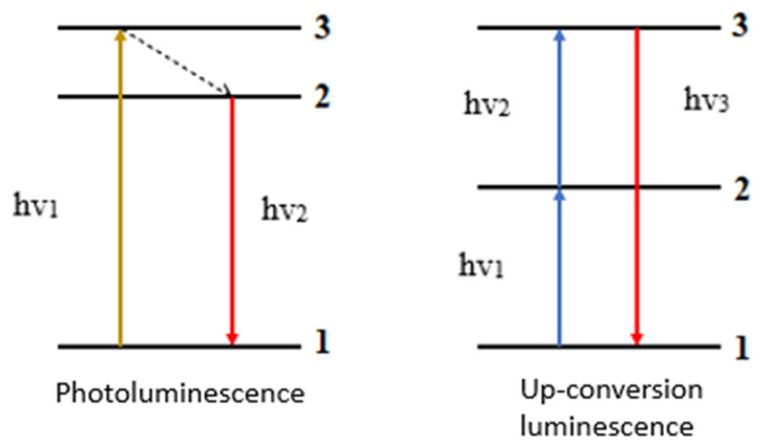
Schematic representation of the processes of classical luminescence and upconversion luminescence.

An important role in nanotechnology, due to the application methods, is played by luminescent materials, e.g., organic dyes or quantum dots. Most of these materials are subject to Stokes’s law: the excitation wavelength is shorter than the emission wavelength, so that a photon with energy lower than the excitation photon is emitted (downshifting, downconversion). The opposite of regular emission is upconversion. It enables the conversion of radiation in the range from infrared to visible and ultraviolet light. Upconversion is responsible for the absorption of two or more photons, thanks to which the emission of radiation with a higher energy than the absorbed energy takes place. This process is also called anti-Stokes emission. Figure 2 presents the differences between photoluminescence and upconversion luminescence [15].

Initial information about upconversion, and in fact about the opportunity to discover and calculate infrared quantum counter (IRQC) in a solid state thanks to detectors, appeared in 1959 [16]. In 1966, Auzel offered an energy-transfer mechanism that takes place between the excited states of ions in the upconversion process [17]. Since then, the interest in upconversion has grown steadily.

Upconversion nanomaterials, thanks to the infrared radiation excitation method, allow for stronger penetration of biological tissues without destroying them. They show high photochemical stability and a high anti-Stokes shift [18]. The lack of background autofluorescence is also important, given the range of excitation used [19].

The phenomenon of upconversion is characteristic of compounds that have the so-called ladder construction with metastable energy levels. This principle is fulfilled by organic compounds, e.g., polycyclic aromatic hydrocarbons, as well as inorganic compounds, e.g., d and f block metal ions.

For the greater part of organic upconversion nanoparticles, the emission of radiation proceeds according to the action of the triplet–triplet annihilation (TTA) mechanism. It consists in exciting the sensitizer to the S_1_ singlet state, then through the interconnection to its triplet state T_1_, from where the energy through the triplet–triplet energy transfer penetrates into the annihilator, also called the emitter, and returns to the S_0_ state itself. When interaction occurs between two excited annihilators in a triplet state, a higher-energy singlet state is induced, from which UC emission begins and the annihilator returns to the ground state S_0_ [20].

The group of inorganic compounds expressing UC is divided into materials based on transition metals (TMs), materials doped with lanthanide ions, materials with Ln^3+^ and TM ions, and semiconductor materials.

Properties of transition metals, which belong to the d block, and the anions belonging to this group containing d-electron metals (VO_4_^3−^, MoO_6_^6−^, TiO_4_^4−^), thanks to the presence of electrons on the d subshell, of the nature of valence electrons, are very dependent on their chemical area. The wide absorption bands are special for these metals, and the chance of nonradiative transitions between their energy states is high, especially at room temperature, which greatly reduces the efficiency of UC processes [21]. However, the great advantage of these compounds is the opportunity to observe tunable luminescence in the near infrared (Cr^3+^, Mn^2+^), their catalytic and magnetic properties. Examples of transition metal compounds exhibiting UC include Cs_2_NaYCl_6_: Mo^3+^, CsCdCl_3_: Ni^2+^, MgCl_2_: Ti^2+^ [22]. Among most promising photoluminescent nanomaterials are UCNP. Those are inorganic crystals that contain ions of trivalent lanthanides. They can convert low-energy radiation into radiation that has shorter wavelength and higher energy [23]. Under infrared (IR) light excitation, UCNPs emit photoluminescence in the visible and IR regions [24]. Additionally, there is a possibility of adding different therapeutic and targeting elements to UCNP surfaces, which makes them useful for many clinical applications [25]. UCNPs have hydrophobic oleate groups on their surface as a result of the synthesis procedure. This causes their instability in aqueous solutions. In order to hydrophilize them, the surface is coated with amphiphilic polymers. One such coating agent is polyethylene glycol (PEG). It ensures colloidal stability and ability to circulate in bloodstream for a long period of time, both of which are required for targeted delivery of nanocomplexes. PEGylation also prevents enzymatic degradation and accumulation in the liver as well as macrophage opsonization [26]. Additional enriching of those particles in molecules capable of selective binding to receptors that are overexpressed on cancer cells is called active targeting. EGFR, VEGFR, integrins, PDGFR, IGF-1R, folic acid receptors, and vitamin receptors are among those particles that might be used as targets. HER-2 is considered to be one of the most important tumor markers, especially in breast cancer but also lung and gastric cancer [27]. A new group of compounds showing UC are nanocrystalline semiconductors (NCs), which are a combination of quantum dots and organic particles [28]. Quantum dots express superior absorption of broad-spectrum radiation, while organic particles only absorb in the near-infrared range, but are excellent emitters. The upconversion mechanism is related to the triplet–triplet annihilation [29]. In the analyzed structures, with the help of semiconductors as photosensitizer, organic molecules as a transmitter and with the help of an annihilator, it is possible to achieve a much higher efficiency of the UC process, even up to 30–40% [30]. An additional advantage of these materials is the possibility of obtaining flexible optoelectronic devices.

Currently, five mechanisms correlated with the upconversion phenomenon can be distinguished: excited state absorption (ESA), cooperative upconversion (CUC), energy-transfer upconversion (ETU), indirect upconversion energy-mediated migration upconversion (EMU), and photon avalanche (PA). Among the processes, upconversion indirect energy migration is usually observed in core–shell structures and upconversion energy transfer [31].

Upconversion energy transfer is created by sequential absorption of photons in a system where there are two different ions, activator and sensitizer, with similar excitation energies and at a short distance from each other. This mechanism is based on the nonradiative energy transfer from the excited sensitizer to the activator, which is also in the excited state. By using the absorbed energy, it is possible to obtain excited state in higher energy. A phenomenon that accompanies this mechanism may be phonon-assisted energy transport, facilitating the occurrence of the process regardless of the energy difference between the ions. ETU also occurs in other ways, such as successive energy transfer, cooperative luminescence or upconversion cross-relaxation. This mechanism is considered to be one of the most effective UC processes, especially for the Yb^3+^/Er^3+^ and Yb^3+^/Tm^3+^ ion pairs [32].

Upconversion indirect energy migration is a mechanism based on energy transfer in shell–core structures, where the photons detected by the sensitizer are transferred to the so-called battery (type II), then through donors (type III) they reach the activator ion that emits radiation. The shell–core structure approves the separation of the ions involved in the conversion process into different regions, as a result of which the mutual extinction of ions is minimized. Another advantage is the transfer of energy between distant ions without energy losses. Such a concept enables the doping of nanomaterials with such ions as Tb^3+^, Eu^3+^ or Dy^3+^ with the content adjusted to a given use (from several to several dozen percent) [32]. The recent goal of UC research is to develop fluorescent dye adsorbed upconversion nanoparticles for excitation of clinically approved photosensitizers for deep-tissue PDT. These fluorescent UCNPs are designed to be biocompatible hybrid materials that can illuminate deep tissue upon exposure to nondamaging power levels of IR light. The amounts of singlet oxygen generated in a cancer cell are also of great importance with regards to realizing real-time dosimetry and an infrared detector will be implemented to detect singlet oxygen produced from the fluorescent nanoparticle-photosensitizer system. Three-dimensional cell cultures can be used to evaluate the tissue depth of PDT upon irradiation with infrared light by sectioning. This research is expected to contribute to the emerging field of nanomedicine by coupling known photodynamic methods with recent advances in nanotechnology to advance the implementation of PDT in cancer treatment.

## 3. Characteristics of UCNPs

**Figure 3:**
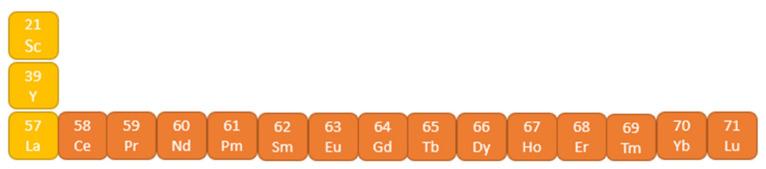
Group of rare earth elements.

Lanthanides are a group of metals from the sixth period of the periodic table, with an atomic number of 58–71 (from cerium to lutetium) Figure 3. They have electrons in their structure on the 4f subshell. The group’s name comes from lanthanum, which, despite the lack of electrons in the 4f subshell, is not a lanthanide species. However, due to their high chemical and physical similarity, along with yttrium and scandium, they are included in the group of lanthanides, forming a group of rare earth elements.

Important features of lanthanides are, among others, ion and atomic beam length, electronic structure of the valence shell, constant oxidation state +3 or paramagnetic properties (except for lutetium).

The electron configuration of the lanthanides [Xe] 6s^2^ 4f^n^ (and 5d^1^ if present) is related to the deposition of the 4f subshell, shielded by filling the 5s and 5p subshell with lower energy. Lanthanide ions in the +3 oxidation state are the most stable among other oxidation states, while some ions can also be at the +2 (eg. Eu^2+^, Yb^2+^) and +4 state (Ce^4+^, Tb^4+^). The most stable electronic structures in the third oxidation state express La^3+^ due to the lack of f electrons, Lu^3+^ with fourteen electrons on the 4f subshell and Gd^3+^, which has a half-filled 4f subshell [33].

Lanthanides are also characterized by contraction; along with an increase in the atomic number, the atomic and ionic radius of a given element decreases. It is a result of the stronger attraction of electrons to the atomic nucleus, which is the result of the increasing number of electrons with a uniform number of electron shells [16].

**Figure 4:**
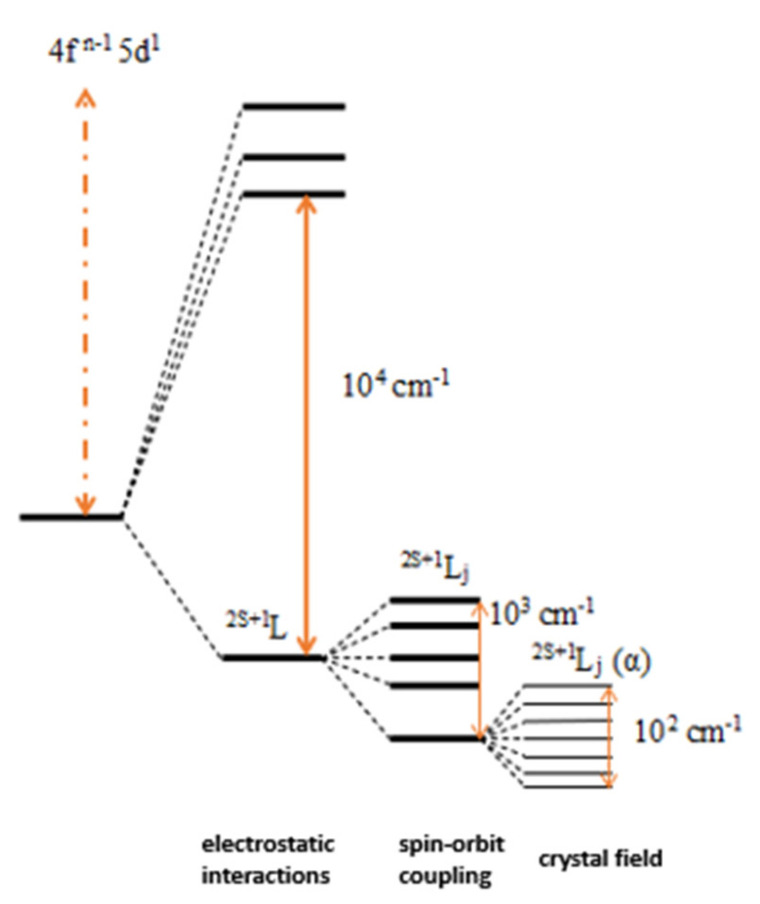
Electronic structure of lanthanide ions. Factors influencing the destruction of levels together with the values of splitting: electrostatic interactions, spin–orbit coupling and crystal field.

The electronic transitions of lanthanide ions are divided into 3 types:

- interconfiguration transitions, nf → (n + 1) d, usually 4f-5d, observed for Pr^3+^ and Ce^3+^ ions, allow transitions, characterized by high intensity in the ultraviolet range,
- charge transfer (ligand-orbital f) transitions, based on the absorption of photons by the ligand and delivering them to the 4f subshell of the Ln^3+^ ion, which will emit the radiation. These transitions are characterized by great spectral width and intensity. They are located in the absorption spectra in the UV range. In addition, they are transitions based on Laporte’s rule (there is a modification of the parity of the electric dipole transitions between energy levels),
- intraconfiguration transitions, f-f, characteristic mainly for Ln^3+^ ions (Figure 4). These are transitions based on Laporte’s rule, yet the bands of these transitions are monitored on the spectra of lanthanides [34].

From among the above types, the most common tests are 4f-4f. The basic factor influencing the lanthanide electrons are electrostatic interactions and less important magnetic interactions, which results in the coupling of the spin and orbital angular momentum of electrons, the so-called spin orbit or Russell Saunders coupling, which expresses the splitting of the energy levels of the 4f subshell electrons into separate multiplets, interpreted by the relationship between the quantum numbers, ^2S + 1^L^J^, where L is orbital, S spin and J is the total angular momentum. As a result of the shielding of the 4fn electrons by the 5s and 5p coatings, the Ln^3+^ ion region has little effect on the splitting of energy levels. The reason is the breakdown of individual multiplets into the so-called Stark levels. The destruction of these levels depends on the symmetry of the environment, e.g., the matrix. The lower the lattice symmetry, the smaller the stratification of the multiplets [35].

Luminescence spectra of 4f-4f electron transitions in Ln^3+^ ions are characterized by narrow bands with a small spectral width. All ions are characterized by an unusual distribution of bands in the emission spectra of a given wavelength; therefore, it is very easy to distinguish ions by means of spectrofluorimetric measurements. In addition, transitions from excited to ground states take up to several milliseconds, which is important in the practical application of compounds with lanthanide ions.

Lanthanides are an ideal material for upconversion due to the well-separated structure of their energy levels and their long service life. In addition, the excited states of Ln^3+^ ions are in the range from UV to infrared, and thus it is possible to observe radiation in a wide spectrum of wavelengths.

UCNPs are constructed using a core of sodium yttrium fluoride (NaYF_4_) co-doped with ytterbium (Yb^3+^) and erbium ion (Er^3+^) coated with an adsorbed fluorescent dye, such as rose bengal. Upon excitation of UCNPs with NIR, FRET-driven fluorescence of the UCNP–rose bengal constructs will be used to excite chlorin-based clinically approved PS, such as photochlor (HPPS), talaporfin sodium (LS11), and mono-L-aspartyl chlorin e6 (NPe6) that have visible light absorptions that overlap with rose bengal florescence. UCNP fluorescence-initiated PDT action of clinically approved chlorin-based PS will be evaluated as a function of depth in 3D cancer cell cultures by sectioning [2,3,4,5,6,7,8,9,10,11,12,13,14]. Several probes and chemical traps can be utilized to detect and quantify the amount of ^1^O_2_ generated by UCNP fluorescence excitation of PS in solution and in cells. The stability of UCNPs under extended irradiation at 980 nm and quantifying the rate of dye photobleaching can be determined. Singlet oxygen-trapping agents can be used to determine the rate of singlet oxygen production.

Inorganic crystals at room temperature show unnoticeable emission or no emission under the influence of excitation in the field of infrared radiation [36]. Hence UC nanomaterials are made of emitting/doping ions, which are the source of the upconversion, and of an inorganic matrix in crystallographic form, providing the correct placement of the luminescent centers. By properly selecting these two components, it becomes possible to control the spectroscopic values of nanomaterials and to select a material with significant properties in a given application [37].

When designing optical systems, such as emission color or quantum efficiency, it is very important to choose the correct UCNP matrix. The most important factor influencing the selection of the matrix is the value of the energy of the crystal lattice vibrations (the smaller the value, the lower the chance of nonradiative relaxation, which is the factor that quenches the upconversion). The physicochemical stability of the matrix is also important. These requirements are met by fluoride matrices, which are characterized by low phonon energy (~350 cm^−1^) and high physicochemical stability, e.g., NaYF_4_ [38]. Bromides and chlorides are also characterized by low energy of crystal lattice vibrations; however, due to their significant hygroscopicity, they have limited applications [39].

Another special feature when designing efficient UCNPs is the matrix crystallographic arrangement and the ion radius of the cations it comprises. An example is the NaYF_4_ matrix, which occurs in the regular and hexagonal system, where upconversion up to 10 times more intense than in the regular system was monitored in a structure with a lower symmetry [40]. This is related to a greater distortion of the crystal field around the emitting ions, which increases the chance of 4f-4f forgotten transitions for Ln^3+^ ions. Another aspect is the ion radius of the cations, which should be approximately the size of the emitting ions, thus preventing the formation of crystal lattice abnormalities during ion exchange, e.g., Ca^2+^ or Na^+^ ions have more or less the same ion radii as lanthanide ions and their inorganic compounds, often are used as matrices [41].

Since inorganic matrices do not participate in the upconversion process, so-called activators, i.e., ions doping matrices, the energy structure of which is built of a large number of metastable excited states with a long lifetime. As a result, activators in the form of lanthanide ions (Ln^3+^) are often used, which have electrons in their structure on the 4f subshell, between which f-f electronic transitions take place. Therefore, for each Ln^3+^ ion having more than one electron on the subshell, a UC phenomenon should be observed. However, only for some ions can the phenomenon of upconversion occur (Er^3+^, Pr^3+^, H^3+^, Tm^3+^, Nd^3+^), of which only Er^3+^, Ho^3+^ and Tm^3+^ register intense upconversion thanks to well-separated excitation states.

The efficiency of the activator is influenced by:

- Concentration in the matrix,
- Cross section for radiation absorption,
- Chance of nonradiative transitions due to the short distance between the excited states [24].

Unfortunately, luminescence lanthanides have a low NIR absorption cross section, which results in a low UC efficiency. It is also not possible to increase the activator content due to the cross-relaxation process of ions placed in close proximity. The highest concentration of impurities, e.g., for the Tm^3+^ ion, is 0.5% and for Er^3+^ 3%. To overcome these limitations, it is necessary to dope the matrix with an additional ion with a high absorption coefficient, which will be a sensitizer, i.e., the NIR radiation absorbing ion, which, by the action of the ETU, efficiently transports energy to the activator ion. Yb^3+^ ions are mainly used for this, due to the simple structure and relatively high cross section for absorption of 980 nm radiation. In addition, Yb^3+^ ions have a weaker tendency to concentration extinction, thanks to which it is possible to multiply the amount of ions in the matrix, even up to 100%. It influences the increase of the molar absorption coefficient of Yb^3+^ ions and the shortening of the distance between the sensitizer and the emitter, increasing the energy transfer [42].

## 4. Capabilities of UC Amplification

Despite the abovementioned possibilities of enhancing upconversion by using Yb^3+^ ions as a sensitizer and optimizing the concentration of impurities in the matrix, the monitored luminescence is still much less efficient than, for example, for organic dyes.

The limitations are [43,44,45,46,47]:

- low cross section for the absorption of Ln^3+^ ions (e.g., Yb^3+^ as a sensitizer shows about 1000–10,000 times smaller cross section for absorption than commonly used organic dyes),
- cross-relaxation processes between ions that are at short distances from each other and the inability to increase the cross section,
- small particles, surface ligands and the medium in which the particles are located,
- low value of the molar radiation absorption coefficient in the NIR range [43].

The nanometric size (high dependence of the NP surface on its volume) leads to damage to the network and the reduction of emissions from the environment. On the other hand, surface ligands and scattering medium are commonly rich in -NH, -OH or -CH oscillators, the vibrational energies of which have similar values to the energy levels of Ln^3+^ ions, and thus they significantly quench the processes of radiant relaxation [48].

However, due to the interesting properties of UCNPs having Ln^3+^ ions, such as high signal-to-noise ratio, lack of background autofluorescence, thin emission and absorption bands, and long lifetimes, scientists are analyzing new synthesis methods and strategies to enhance the luminescence of UC nanomaterials,

The most common strategies to enhance upconversion are the prudent design of materials expressing this process and the integration of UCNPs with other materials.

**Figure 5:**
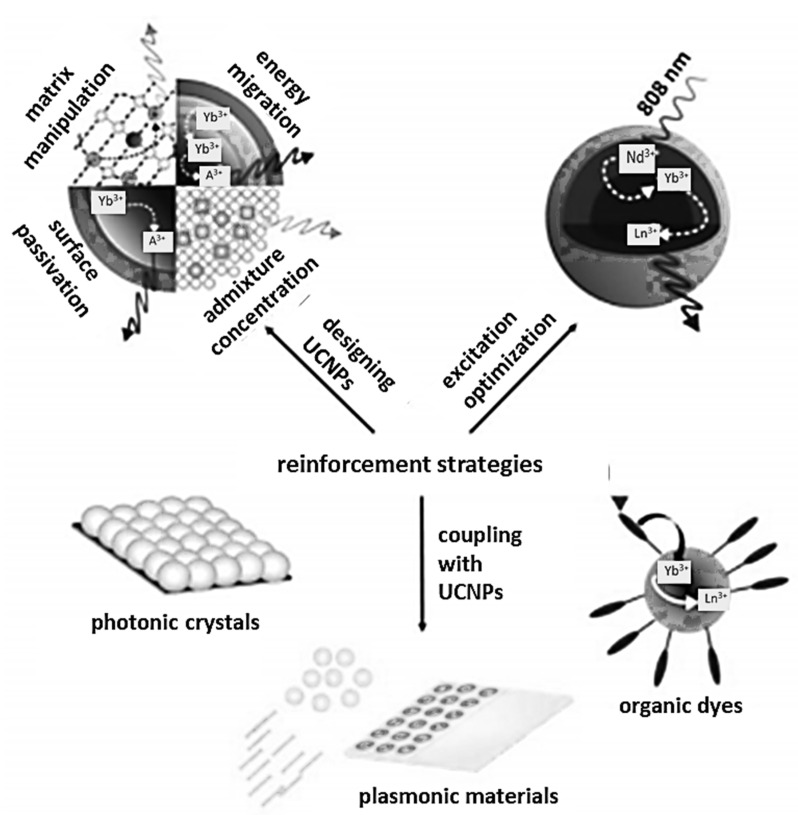
Upconversion enhancement strategy.

The first strategy includes the correct selection of the matrix and the emitting ions, their concentration, passivation of the particle surface, design of the core–shell structures, and optimization of energy transfer between ions and upconversion excitation. The second type of upconversion enhancement consists in combining emission-sensitive nanoparticles with organic dyes, surface plasmons and photonic crystals (Figure 5).

The addition of Li^+^, Na^+^ ions, or transition metals, e.g., Bi^3+^, Sc^3+^, Fe^3+^, Zn^2+^, may enhance the observed luminescence by increasing the asymmetry of the crystallographic structure. This may contribute to the partially allowed nature of the electronic transitions due to mixing of the 4f levels with the energy levels of the additional ions (e.g., d-f). Another effect affecting the increase in luminescence by additional doping with the mentioned ions is, among others, an increase in the electron–photon coupling and a decrease in the size of the unit cell [49].

Prudent creation of nanomaterials presupposes low concentrations of emitting ions due to cross-relaxation processes. It is interesting that the use of high laser power (over the energy causing the saturation effect, even 5 × 10^6^ W cm^−2^) may partially eliminate the extinction of the upconversion with the use of high Ln^3+^ ions and affect the modification of the emission color and the photonicity of the process. The efficiency of UC can also be influenced by the excitation of several wavelengths at once [50].

Another method of improving the efficiency of the upconversion is to use an excitation wavelength different from the commonly used 980 nm. The advantage of this approach is the reduction of the heating effect of the cells, which results from the high absorption of 980 nm radiation with water, which is their main component. To solve this problem, the excitation wavelength needs to be shortened by using Nd^3+^ ions, thanks to which radiation with a wavelength of around 808 nm is absorbed. Importantly, Nd^3+^ can act as an activator and sensitizer [51]. The matrix is also doped with Yb^3+^ ions together with Nd^3+^ and the emitter ion, but due to the cross-relaxation process between ions, the concentration of Nd^3+^ cannot exceed 3%. To avoid this limitation, the assumption of a cascade energy transfer was created, in which Nd^3+^ ions would be separated from the emitter ions in the active core–active shell structure (NaYF_4_: Nd^3+^/Yb^3+^/Er^3+^ @ NaYF_4_: Nd^3+^/Yb^3+^) offered by Huang and Lin [52].

The creation of core–shell structures allows for the enhancement of the observed luminescence by obtaining rational energy transfer between ions, transforming the excitation wavelength and surface passivation [53].

The second UC amplification strategy is based on the combination of inorganic nanoparticles containing lanthanide ions with surface plasmons, photonic crystals and organic dyes.

Surface plasmons can be created thanks to the irradiation of the metals and semiconductors their irradiation, as a result of which the released electrons accumulate on the surface of the material, where they form surface plasmon resonance by oscillation. Typically, metals such as gold and silver in the form of coatings, thin films or metallic nanoparticles are used to enhance absorption and upconversion emissions [54].

Photonic crystals are dielectric optical materials with a systematically changing refractive index, thus affecting the movement of photons. Due to the alternating occurrence of high and low refractive index layers, some waves are transmitted and some are reflected. Photonic crystals are capable of influencing the emission wavelength, intensity and direction. To enhance the absorption and upward conversion emission, crystals based on inverted silica, opal, cadmium sulfide or silicon nitride are used [55].

Significant enhancement of upconversion is also observed when UCNPs are combined with organic molecules, and more precisely with organic dyes, which have in their structure systems of conjugated π bonds (so-called chromophores). They enable a much greater absorption of radiation in a wide range, characterized by up to 10,000 times greater cross section for absorption than Ln^3+^ ions [56]. In addition, it is permissible to change the excitation wavelength, e.g., 808 nm, thanks to which it is possible to reduce the heating effect of the system [50].

## 5. Examples of UCNP Synthesis 

### 5.1. Synthesis of NaYF_4_ Yb^3+^Er^3^ Nanoparticles

Synthesis of the UCNP core is accomplished by published procedures [1,2,3,4,5,6,7,8,9,10,11,12,13,14]. Briefly, (0.8 mmol) and 0.2 mmol YbCl_3_ and 0.003 mmol ErCl_3_ were mixed with 6 mL of oleic acid and 15 mL of octadecane in a 50 mL flask and heated to 160 °C to form a homogeneous solution. After cooling to room temperature, 10 mL of methanol solution containing 2.5 mmol NaOH and 4 mmol NH_4_F was added into the flask and stirred for 30 min. The solution was heated to remove methanol, degassed at 100 °C for 10 min followed by heating to 300 °C and cooling under argon. After cooling to room temperature, the oleate-capped nanocrystals were precipitated with ethanol, washed with 1:1 ethanol:water and stored in hexane. UCNP batches in hexagonal phase with sizes of 20–30 nm as measured by transmission electron microscopy were used to adsorb fluorescent dye.

### 5.2. Replacement of Oleate with Rose Bengal

**Figure 6:**
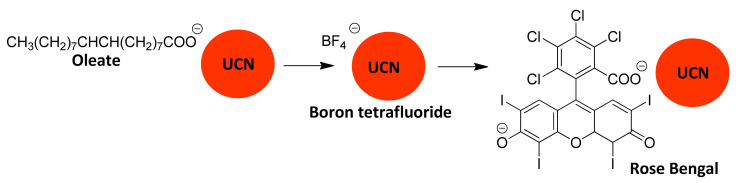
Nitrosyl tetrafluoroborate mediated ligand exchange to replace oleate with fluorescent rose Bengal.

Oleate on the synthesized UCNPs can be replaced with the fluorescent dye rose bengal by a nitrosyl tetrafluoroborate (NOBF_4_)-mediated ligand exchange reaction [1,2,3,4,5,6,7,8,9,10,11,12,13,14]. UCNPs with oleate cap are stirred in a two-phase hexane/DMF (or hexane/DMSO) solution and as oleate is replaced by BF_4_^−^, the UCNPs move from hexane to the more polar DMF (or DMSO) phase. Stirring the BF_4_^−^ coated UCNPs with rose bengal leads to a second exchange (Figure 6).

### 5.3. Fluorescence Emission and Absorption Quenching of ^1^O_2_ Probes

There are several probes that can be utilized to detect and quantify the amount of ^1^O_2_ generated by fluorescent UCNP excitation of PS in solution and in cells that rely on absorption decay or increase of probe fluorescence emission. Fluorescence probes are useful for confirming the presence of ^1^O_2_ and approximating concentration. Real-time dosimetry is more challenging and requires direct measurement of ^1^O_2_ luminescence at 1270 nm.

### 5.4. Extracellular Measurement of ^1^O_2_ [14]

Singlet oxygen sensor green (SOSG or trans-1-(2’-methoxyvinyl) pyrene) generates chemiluminescence upon decomposition to 1-pyrenecarboxaldehyde at 465 nm. UCNPs (0.5 mg mL^−1^) and photosensitizer (1.0 µM) will be dispersed in 5 mM SOSG aqueous solution. Irradiation at 980 nm at several laser excitation intensities (0.5–10 Wcm^−2^) will generate oxidized SOSG. Immediately following irradiation, the laser is turned off and the emission of oxidized SOSG at 465 nm is measured by a UV-vis spectrophotometer. SOSG is cell-impermeant and can be used either in the presence of model cells or in aqueous solution to approximate concentrations of extracellular ^1^O_2_ generated.

### 5.5. Measurement of ^1^O_2_ in the Absence of Cells: [14]

The singlet oxygen probe 1,3-diphenylisobenzofuran (DPBF) was utilized. UCNPs (0.5 mg mL^−1^) and PS (1.0 µM) were dispersed in neat DPBF solution (3 mL). Irradiation at 980 nm at several excitation intensities (0.5–10 Wcm^−2^) yields oxidized DPBF. Immediately following irradiation, the laser is turned off and the absorbance of DPBF is measured at 410 nm by a UV-vis spectrophotometer. Several experiments will generate a time-course graph of the decrease in absorbance of DPBF at 410 nm. A particularly useful probe to measure ^1^O_2_ in water is 9,10-anthracenediyl-bis(methylene)dimalonic acid (ABDA). Similarly to DPBF, the absorbance of ABDA at 378 nm decreases when it reacts with ^1^O_2_. Water solubility of ABDA may also be increased if needed by increasing the pH of the solution, which can partially or fully deprotonate ABDA. The cell-permeable dye 2’,7’-dichlorofluorescin diacetate (DCFDA) can be used to confirm the presence of ROS intracellularly. Fluorescence of the oxidized products of DCFDA confirms the presence of ROS aspecifically and would not be a specific test for the presence of ^1^O_2_. The cell-permeable dye 2’,7’-dichlorofluorescin diacetate (DCFDA) is deacetylated inside the cell to a nonfluorescent compound that reacts with ROS to form 2’,7’-dichlorofluorescin (DCF), which can be detected by flow cytometry by excitation at 495 nm and emission at 529 nm. In a typical procedure, UCNPs and PS were both incubated with cells for 6 h in the dark. Cells are then washed with phosphate-buffered saline (PBS) to remove uninternalized UCNPs or PS. Fresh cell media containing 20 μM DCFDA are added to cells in the dark and incubated for 1 h. Cells containing UCNPs. PS, and DCFDA are irradiated at constant power at 980 nm for 30 min. Irradiated cells are collected by trypsinization, resuspended in PBS and filtered through 35 mm nylon mesh. The amount of intracellular ROS is determined by measuring DCF by flow cytometry with an excitation wavelength of 485 nm and emission at 525 nm.

UCNPs are constructed using a core of sodium yttrium fluoride (NaYF_4_) co-doped with ytterbium (Yb^3+^) and erbium ion (Er^3+^) coated with an adsorbed fluorescent dye, such as rose bengal. Upon excitation of UCNPs with NIR, FRET-driven fluorescence of the UCNP–rose bengal constructs is used to excite chlorin-based clinically approved PS, such as photochlor (HPPS), talaporfin sodium (LS11), and mono-L-aspartyl chlorin e6 (NPe6) that have visible light absorptions that overlap with rose bengal florescence. UCNP fluorescence initiated photodynamic action of clinically approved chlorin-based PS is evaluated as a function of depth in 3D cancer cell cultures by sectioning [1,2,3,4,5,6,7,8,9,10,11,12,13,14].

## 6. Applications of UCNPs

Due to the unique properties of UCNPs doped with lanthanide ions (considerable anti-Stokes shift, thin bands in the excitation and absorption spectra, infrared excitation, photochemical stability, material abrasion and lack of background autofluorescence) they are used in many fields of science and in everyday life.

A significant group of UCNPs is used in advanced technologies or optoelectronics (e.g., semiconductor lasers, solar cells, fiber optic amplifiers, optical waveguides, 3D displays) [56]. More and more research is also carried out in the area of securities, documents, medicines and in forensics (fingerprints). You and others presented a fascinating application of using UCNPs based fluorescent QR codes using three colors (red, green and blue, RGB) to label drug capsules with the opportunity to discover information about it after scanning the code in a designated application [57]. Inorganic upconversion nanomaterials are also used in photoisomerization and photocatalysis reactions [58].

UCNP materials have a wide analytical application for the detection of specific pH, metal ions, temperature, anions, biomolecules and free radicals [59]. Mainly, the detection takes place as a result of resonant energy transfer (FRET or LRET), where the test substance is an acceptor and nanoparticles are the energy donor. Due to the presence of a given substance in the analyte, modifications in the absorption spectra or differences in the distance between the donor and acceptor are monitored [60].

**Figure 7:**
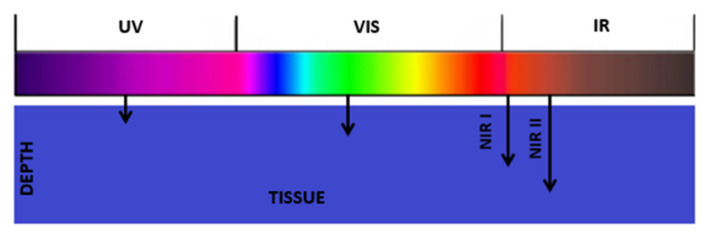
Diagram of tissue penetration by radiation of unequal wavelengths.

Most research with the use of UCNPs is carried out in biology, medicine and related sciences, thanks to the existence of the so-called optical window. There are three optical windows, the first (NIR I, 750–900 nm), the second (NIR II, 1100–1350 nm) and the third (NIR III, 1500–1700 nm) [61]. When using excitation in these ranges, intensive tissue penetration is possible (the longer the wavelength, the more intense the penetration, (Figure 7), with reduced photon scattering and negligible autofluorescence. In addition, considering the use of infrared radiation, the detrimental effect on healthy cells is much smaller compared to ultraviolet excitation.

An example of the use of UCNPs in biology and medicine is mainly bioimaging (e.g., luminescence of upconversion, combination of luminescence of upconversion with the technique of computed tomography or magnetic resonance, contrast agents), cell therapies (photothermal and photodynamic therapy), the abovementioned pH sensors, temperature and biomolecule diagnostic tests, drug-delivery monitoring and others [62].

Another interesting technique is optogenetics, which uses light to stimulate and control neurons. UCNPs introduced into the body can inhibit/stimulate given neurons due to blue or green luminescence induced by NIR radiation [63].

Imaging techniques such as stimulated emission depletion (STED) and 3D imaging are also developing well: 3D imaging of cells based on UCNPs is possible by confocal microscopy, thanks to which images with high contrast and resolution are obtained. The successor of confocal microscopy is STED, which produces high-resolution images by overcoming the diffraction limit. Due to the lack of the observed photobleaching of UCNPs, unlike the organic dyes used in this technique, nanoparticles with Ln^3+^ ions are optimistic about STED microscopy [64].

MTT test used for cytotoxicity testing showed that UCN-DARPin-LoPE reduced the service life of SK-BR-3-Kat cells overexpressing the HER2 receptor. In contrast, in HER2-negative CHO cells no significant cytotoxic effect was observed. Blocking the protein synthesis due to the action of the target toxin DARPin-LoPE caused the elimination of cancer cells in HER2-positive cancer cells [65].

Farka et al., in his work compared three types of streptavidin–UCNP conjugates to conventional fluorescent labeling. In his study, PEG-coated UCNPs showed lower levels of aspecific binding than BSA-coated labels (SA-BSA-UCN). Additionally, a blocking buffer composition without serum proteins decreased the level of aspecific binding. BT-474 cells positive for HER2 showed the highest specific signals after SA-PEG-Ner-UCNP labeling and extremely low background interference that was observed by wide-field upconversion microscopy and upconversion scanning (S/B319). Conventional fluorescent labeling has only reached S/B6. Therefore, the optimized SA-PEG-Ner-UCNP labeling protocol resulted in a 50-fold wider score dynamic range than fluorescence labeling [66].

Panikar et al., in his article published in 2019 announced that he used ligand-targeted nanoliposomes for encapsulating doxorubicin (DOX) for chemotherapy, and methylene blue (MB) attached NaYF_4_:Yb,Er upconversion nanoparticles UCNPs for NIR-activated bioimaging and enhanced PDT. They observed a 95% decline in cell viability using chemo-photodynamic combinational therapy. In contrast in chemotherapy the proliferation declined by 77% and in PDT by 84% [67]. Ramirez-Garcia et al., in their study covered UCNPs with a photocatalytic TiO_2_/ZrO_2_ coating. As a result, they achieved greater ROS production and emission band focused at 801 nm, which is useful for tracking cells at tissue depth level. Bioconjugation with the monoclonal antibody trastuzumab guaranteed specificity for HER2-positive breast cancer. In their study, a decrease of 88% in cell viability was achieved by this change [68]. In further studies, it has been shown that theragnostic nanoconjugate is an alternative to HER2-positive breast cancer imaging and selective photodynamic therapy. Trastuzumab ensured the specificity to HER2-positive cells and enabled the UCNP-ZnPc-Tras to selectively reduce the viability of breast cancer cells to 21% after 5 min of exposure to infrared light. In the absence of IR light those particles were practically inactive [69]. Nanophosphors are different type of contrast agents. They are used for deep-tissue imaging since they can be excited by X-ray and NIR light. Chen et al., in his article from 2017 described a new method using X-ray nanophosphors (Gd_2_O_2_S:Eu or Tb) and upconversion nanophosphors (Y_2_O_2_S: Yb/Er, or Yb/Tm) with large crystal domain without aggregation. A protective shell was made by restricting NaF only to the core. Those particles were then coated with PEG–folic acid so that they could target MCF-7 breast cancer cells that overexpress folic acid receptors. This protective shell increased luminescence intensity of X-ray (Gd_2_O_2_S:Eu or Tb) and upconversion (Y_2_O_2_S:Yb/Er or Yb/Tm) nanophosphors more than 10 times in comparison to particles without sintering agent. Additional coating with PEG-FA increased cellular uptake by breast cancer cells [70].

Another idea of nanoparticles was presented by Han et al. They covalently crafted UCNP with chitosan-functionalized MoS2 (MoS2-CS) and Folic acid (FA) and then loaded phthalocyanine (ZnPc) on the surface of MoS2. This integrated PDT with photothermal therapy (PTT) and luminescence imaging for greater efficiency. The researchers obtained greater therapeutic result thanks to the synergistic effect of both treatments [71].

Multidrug resistance (MDR) of cancer cells is a huge clinical problem, especially in terms of treatment. Pan et al., presented a multifunctional delivery system based on DOX-encapsulated NaYF_4_:Yb/Er@NaGdF_4_ yolk–shell nanostructures for managing chemotherapy and dual imaging of drug-resistant breast cancer. The research showed good biocompatibility of those materials, good stability, and the delivery and uptake of DOX were greater in drug-resistant cells. Additionally, the scientists obtained a composite that might be used as an imaging probe. This could be used for dual-modal imaging and enhanced chemotherapy in patients with MDR breast cancer [72].

A different use of UCNPs was presented by Gallo et al. In his article he described chelation of radionuclides, 68Ga, to MRI/optical particles. This enabled the particles to be used as both MRI contrast agents (DO3A-Gd^3+^) and PET tracers (DO3A-68Ga^3+^). Clinical application includes diagnosis of cancer and imaging, which might be useful especially for surgeons preparing the patient for operation [73].

Another clinical aspect of breast cancer is the search for metastases that is crucial in planning the treatment. Qui et al., used a probe made with NaGdF_4_:Yb,Tm,Ca@NaLuF_4_ core–shell upconversion nanoparticles bound with anti-HER2 antibodies. Applied to mice, it allowed detection of lymph-node metastases of breast cancer. Additionally, surface antibodies changed the half-life of nanoparticles, making them more resistant to elimination [74].

Apart from the HER-2 receptor in breast cancer cells, researchers evaluated the potential clinical use of Y1 receptor ligand combined with UCNP enriched with photosensitizer. Yu et al., synthesized Y1Rs ligand [Pro30, Nle31, Bpa32, Leu34] NPY(28-36) (NPY) modified and photosensitizer MC540 loaded LiLuF_4_:Yb,Er@nLiGdF_4_@mSiO_2_ multifunctional nanocomposites with core–multishell structure. They researched two main parameters: PDT therapeutic efficacy and toxicity. They showed that their nanocomposites may serve as a very good theranostic agent with clinical potential in diagnosing and therapy of breast cancer overexpressing Y1 receptor [75].

## 7. UCNPs Toxicity

Increasingly, nanoparticles containing Ln^3+^ ions require consideration of their impact on living organisms and the natural environment, including decomposition, toxicity and degradation. Due to the short period of research on UC nanomaterials, it is difficult to predict the impact of UCNPs on ourselves and the environment in the long run. Therefore, studies showing the interaction of nanoparticles with living organisms and inanimate matter are special [76].

The toxicity of nanoparticles may be influenced by their shape, size, charge and surface transformation, product purity, element composition and their stability in water. Despite conducting in vitro tests, it is hard to say how nanoparticles can behave in organisms with a more complex structure. However, current studies show negligible cytotoxicity of UCNPs based on lanthanides. According to Li et al., nanoparticles NaYF_4_: Yb^3+^, Tm^3+^, Gd^3+^ @ PEG after injection into mice, after 1 h accumulate mainly in the liver and spleen, after 24 h they accumulate in the kidneys, decreasing in the liver and spleen, and after 30 days a small amount is in the lungs. In addition, the impact of nanoparticles on kidney and spleen cells was recorded, and no negative effect was noted [77].

However, it has been noticed that it is possible for nanoparticles to generate reactive forms of oxygen (ROS), which may negatively affect the processes performed in cells and cause their damage. However, ROS or singlet oxygen (^1^O_2_) is capable of wiping out disease cells in the body, e.g., cancer cells. Correct combination of nanoparticles with a photosensitizer (e.g., zinc phthalocyanine, rose bengal or methylene blue) that absorbs radiation emitted by NPs and then produces ROS, directs reactive oxygen species to damaged cells [78].

The stability and durability of nanoparticles are also important properties. The possibility of dissolving the material in water can release fluoride, which, when accumulated in the body in excess, contributes to the disturbance of many enzymatic processes. In addition, lack of sufficient stability may contribute to the release of lanthanide ions. Another problem may be the frequent aggregation of nanoparticles accumulating in organs, where macrophages can cause their decomposition, and adolescent compounds may negatively affect the processes taking place in cells and themselves. The formed aggregates can also obstruct the natural blood flow [79].

UC nanomaterials containing Ln^3+^ ions are widely used in biology and medicine, but it is important to carefully study the unwanted effects that may develop in organisms during their use. Despite in vivo and in vitro studies showing a slight toxicity of NPs, the physicochemical studies of UCNPs as well as their interactions with tissues, cells and complex living organisms are important. Without detailed tests and analyses of the influence of nanoparticles, especially during long-term interaction with organisms, their safe use is unrealistic.

## 8. Discussion

The combination of the characteristic physicochemical and optoelectronic properties of UCNPs with biological activity makes it possible to obtain highly specialized equipment in the fields of diagnostics and therapy. It is possible to design tools of this kind when the NPs are properly adapted to obtain a hydrophilic surface, biocompatibility and low toxicity. In order to obtain the abovementioned properties, nanoparticle-synthesis procedures and surface modification are used. Most often, organic solvents are used to obtain the presence of hydrophobic ligands on the NPs surface [53].

There are several methods used to modify NP surfaces. The most commonly used methods include:

- removal, exchange, oxidation and absorption of a ligand (a compound capable of binding to a receptor protein),
- silanization and layer deposition on the particle surface [53].

Modifying the surface of nanoparticles by removing ligand is achieved by treating NPs on the surface (e.g., with oleic acid) with hydrochloric acid in an acidic environment, which causes its detachment. Then, the surface of the nanoparticles requires the addition of electronegative groups (-SH, -COOH, -NH_2_ and -OH) to facilitate bioconjugation to the molecules. Tertrafluoroboranunitrosone (NOBF_4_) in dimethyl sulfoxide (DMSO) or dimethylformamide (DMF) is also used to remove the ligand [53].

Ligand absorption is comparable to its efficient method as its removal. In the case of this method, there are van der Waals interactions between the hydrophobic parts of the original and the new ligand (the most commonly used amphiphilic polymers). The hydrophilic end in this case improves the affinity of the particles for water, which enables the formation of stable aqueous colloids. Various polymer structures are used, e.g., poly (L-lysine) (PLL), 6-aminohexanoic acid (6AA), polyacrylic acid (PAA), polyethylene glycol and polycaprolactone copolymer (PEG-block-PCL), polymaleic anhydride-alt-1-octadecene (PMAO), octylamine-polyacrylic acid-polyethylene glycol (OA-PAA-PEG) [54].

Another equally popular modification method is the coating of nanoparticles with a silica coating and surface silanization. NPs are covered with a thin layer of silica, which is modified with hay derivatives (rich in amine, carboxyl or thiol groups). These changes may be carried out simultaneously or sequentially.

The method of modification with the use of silica has many advantages:

- the silica layer is transparent,
- is atoxic,
- biocompatible,
- has no major influence on the optical properties of the material,
- can be used for hydrophobic and hydrophilic materials [55].

Silanization is carried out using the Stöber method and reverse microemulsions. Converting photoluminescence for the application is an interesting feature that can be used in a variety of fields. In bioimaging, upconversion is particularly advantageous because it allows the use of low-energy infrared light capable of penetrating deeper into biological tissue than UV light and causes less tissue damage. Innovative 3D volumetric display technologies treat UCNPs as photoactive elements that-finely dispersed in a transparent volumetric matrix can be switched from “off” to “on” using a set of IR lasers that can create true, full-color 3D images. Dye-sensitive solar cells (DSSCs) are another application of lanthanide-based materials that is currently extensively researched. The fascinating ability of lanthanide-based materials to upconvert from near-infrared to visible light makes them ideal nanomodifiers for photovoltaic devices that are usually limited to visible-light conversion. In addition to the technique of deposition of lanthanide ions in nanoparticles, such as NaYF_4_, they can also be complexed with organic compounds, which in turn can be attached or embedded in an inorganic or organic matrix. Also, cellulose nanofibrils with surface carboxyl groups can be used as such a complexing matrix to produce photoluminescent materials.

One specific limitation of current methods of photodynamic therapy is that the visible light used to activate photosensitizer has a shallow tissue-penetration depth of several millimeters. This limits the application of photodynamic therapy to surface cancers in the absence of a technique to illuminate deeper tissue. Fiberoptic endoscopy can aid in the delivery of visible light to remote anatomical sites within the body that cannot be illuminated with an external light source. Another approach is to use nanoparticles which can emit the visible light needed to activate photosensitizer. Infrared light, as opposed to visible light, can penetrate tissue to a depth of several centimeters but cannot activate visible-light absorbing photosensitizers. Biocompatible nanoparticles can be designed to absorb infrared light and emit visible light in a process called upconversion. Upconversion is a process whereby matter absorbs long wavelength light such as infrared and emits visible light. Fluorescent dyes can be adsorbed to upconversion nanoparticles and photodynamic therapy can be achieved in deeper tissue upon irradiation with infrared light. Fluorescent upconversion nanoparticles also afford selectivity and control as they may be activated only by illumination of an area of diseased tissue, such as a tumor, with infrared light and are by themselves atoxic in the absence of infrared light. Also, fluorescent upconversion nanoparticle constructs can be monitored as to their location in the body and uptake by cancer cells, aiding in evaluation of exact doses required to treat the targeted cancer. One approach to cancer treatment that is increasingly gaining in popularity for clinical use is photodynamic therapy. PDT is a treatment that uses a combination of light-absorbing photosensitizer and dissolved oxygen to kill cancer. As a cancer treatment, photodynamic therapy is considered to be a milder approach, as oxygen is the active agent responsible for achieving cancer cell death as opposed to agents that are toxic to the body as a whole, such as chemotherapy drugs and radiation. Additionally, the light-absorbing photosensitizers are atoxic to the body in the absence of light. Photodynamic therapy is also a method that can be controlled with regards to the location of the treatment as it requires selective illumination of the treatment area with light. Selective illumination and light activation then enable selective damage of diseased tissue without damaging the surrounding healthy tissue. For example, if light-absorbing photosensitizer is injected into a patient, only the tumor needs to be illuminated with light which initiates the cancer cell-killing effect. Several photoactivated photosensitizers have been approved for clinical use in the treatment of cancers such as basal cell carcinoma, early lung cancer and malignant skin tumors. The treatment of cancer continues to be a challenging task and the combination of medicine with nanotechnology can advance photodynamic therapy by enhancing control with regard to specific treatment location and activation by sources of illumination in deep tissue. One specific limitation of current methods of PDT is that the visible light used to activate photosensitizer has a short tissue penetration depth of several millimeters. This limits the application of photodynamic therapy to surface cancers in the absence of a technique to illuminate deeper tissue. Fiberoptic endoscopy can aid in the delivery of visible light to remote anatomical sites within the body that cannot be illuminated with an external light source. Another approach is to use nanoparticles that can emit the visible light needed to activate photosensitizer. Infrared light, as opposed to visible light, can penetrate tissue to a depth of several centimeters but cannot activate visible-light absorbing photosensitizer.

## 9. Conclusions

Förster resonance energy transfer (FRET)-driven fluorescence from dye-adsorbed lanthanide upconversion nanoparticles has the potential to extend the depth of photodynamic therapy (PDT) by transducing tissue-penetrating near-infrared (NIR) light to visible light that can be absorbed by PDT photosensitizers. The impact of this research will be to broaden the scope and advance PDT cancer treatment. We expect that the results from synthesis and evaluation of FRET-driven fluorescent upconversion nanoparticles will serve to advance understanding of the factors necessary to achieve deep-tissue photodynamic therapy. Undoubtedly, this research will be a starting point to understanding how upconversion may broaden the scope of photodynamic therapy with the use of infrared light and will lead to a greater understanding of the robustness of nanoparticle constructs and cell-targeting mechanisms. FRET-driven fluorescent upconversion nanoparticle excitation of PS has great potential in next-generation photodynamic therapy modalities.

## Data Availability

Data are contained within the article.
